# A comparative analysis of disability measures in Cameroonian surveys

**DOI:** 10.1186/s12963-019-0198-4

**Published:** 2019-12-05

**Authors:** Arlette Simo Fotso, Géraldine Duthé, Clifford Odimegwu

**Affiliations:** 1 ICAP at Columbia University, New York, USA; 2 Demography and Population Studies, Schools of Public Health and Social Sciences, University of the Witwatersrand, Johannesburg, South Africa; 3 Institut National d’Études Démographiques (INED), Paris, France

**Keywords:** Disability, Impairment, Activity limitation, Participation restriction, Measure, Cameroon, Confidence intervals, Association

## Abstract

**Background:**

Although identifying vulnerable groups is an important step in shaping appropriate and efficient policies for targeting populations of disabled people, it remains a challenge. This study aims to evaluate for the first time the comparability of the different disability measurements used in Cameroon. This is done by comparing them with the international standards proposed by the Washington Group (WG). It also evaluates the consistency of the association between the disability as measured by these surveys and the sociodemographic characteristics.

**Method:**

We used data from the third Cameroonian Population and Housing Census (3RGPH) of 2005, the third Cameroonian Household survey (ECAM3) of 2007, the Demographic Health and Multiple Indicator Cluster Survey (DHS-MICS) of 2011 and a survey conducted on adults in Yaoundé (HandiVIH) in 2015 with the WG tool. The proportion and their confidence intervals, chi-square tests and multivariate logistic regressions are used for analyses.

**Results:**

In the city of Yaoundé and for the 15–49 age group, disability prevalence was estimated at 3.6% (CI = [2.5, 5.1]), 2.7% CI = [2.1, 3.5]), 2.6% (CI = [2.4, 2.7]) and 1.0% (CI = [1.0, 1.10]), according to DHS-MICS, ECAM3, HandiVIH and 3RGPH, respectively. The prevalence of severe motor and mental disabilities in DHS-MICS (0.4% CI = [0.2, 0.8], 1.1% CI = [0.7, 1.8] and 0.5% CI = [0.2, 1.1], respectively) are not significantly different from the findings of HandiVIH (0.3% CI = [0.2, 0.3], 0.8% CI = [0.7, 0.9] and 0.5% CI = [0.5, 0.6], respectively). Only motor disability prevalence in ECAM3 (0.8%, CI = [0.5, 1.2]) is not different from that of HandiVIH. When the WG screening tool is used in HandiVIH, disability is positively associated with age, negatively associated with educational level, being in a union and socioeconomic status (SES) and it is not associated with sex. Severe disability, for its part, is not associated with SES and is positively associated with being a male. A different association trend is observed with 3RGPH, ECAM3 and DHS-MICS.

**Conclusion:**

None of the instruments used in the nationally representative Cameroonian surveys produced both disability prevalence and association trends that are exactly similar to those obtained when using the WG disability screening tool, thus highlighting the necessity to include the WG questions in nationally representative surveys.

## Background

Understanding the needs of vulnerable populations is an important step in shaping appropriate and efficient policies for targeting these populations. However, this is only possible if these groups can be clearly identified. It is a real challenge when it comes to disabled people who live in low- and middle-income countries (LMIC), largely due to (1) a global scarcity of health data, and (2) the challenge of measuring disability among the general population. This situation leads to difficulties in providing a clear picture of disability prevalence. In Cameroon, for example, this prevalence was estimated at 1.8% among adults aged 25–59 years in 2005 [1] and at 4.1% among adults aged 15–64 years in 2007 [2]. For children, disability prevalence was evaluated at 22% in the age group 2–9 years old in 2006 [3], whereas only 1% of children under 15 were identified as disabled according to the 2005 census [1]. In the Northwest Region of Cameroon, disability prevalence was estimated at 6.2% in 2010 [4] while it was 10.5% for the same region in 2014 [5].

While part of the difference could be attributable to the year of the survey, the age group and the area delimitation, we also assume that a significant part is attributable to the screening instrument. Indeed, when data are available, various screening instruments are used in household-based surveys. Many surveys are not specifically devoted to disability or even to health (such as population censuses) and they do not necessarily use any instrument that has been validated by the international scientific community.

The common framework for defining disability was established by the World Health Organisation (WHO) through the International Classification of Functioning, Disability and Health (ICF) [6]. This classification is based on the biopsychosocial model, which is a synthesis of the medical model and the social model. The medical model considers disability to be an aspect of a person that requires medical intervention or treatment, while the social model views it as a socially created problem in need of a political response.

According to the ICF framework, disability can encompass three dimensions [6]. The first dimension is impairment; this can be defined as the limitation of a body function or structure. The second one, activity limitation, is related to limitations in performing specific tasks or activities. The last one is participation restriction. This dimension refers to the limitations and restrictions that people experience in their daily lives. Depending on how the disability screening questions are formulated, a survey can capture one, some, or all aspects of one’s disability.

The Washington Group on Disability Statistics (WG) is a United Nations (UN)–sponsored city group commissioned to improve the quality and international comparability of disability measures. To this end, they developed a set of disability measurements and in 2006 proposed a short set of disability measures comprising six core functional domains: seeing, hearing, walking, cognition, self-care and communication. In 2009, an extended version was developed and since 2011 they have been developing a version for children. However, these measures are rarely used in African countries.

Disability measurements have been subject to numerous analyses. Some authors have criticised the self-reported measurement of disability, especially the one related to work limitation, stating that it can be subject to justification bias [7]. The justification bias occurs if the person expects that the screening process could result in some disability benefits or if they want to justify poor economic situations. These authors have advised that measurements of specific health conditions and disabilities (through questions about impairment, activity limitation or clinical screening) should in general provide more objective measures. However, several studies have criticised the instruments that aim to screen impairment only [8]. One of these criticisms concerns the direct question that uses the words “disability” and/or “handicap”.

The word “disability” is often associated with stigma [9]. Measurement errors may also occur because people can be unaware of their disability status or be incorrectly diagnosed, especially in developing countries [8]. In the Northwest Region of Cameroon, a study by Cockburn et al. [4] conducted in 2010 screened for disability by asking the head of the household, “Is there anyone in the house who has any form of disability or handicap?”, which was followed by a checklist from the International Classification of Functioning, Disability and Health (ICF) for any household members who were reported being only disabled [10]. They found a 6.2% prevalence of disability. Furthermore, nearly 90% of those who screened positive for disability had a participation restriction or activity limitation that was moderately severe or greater, and for this reason the authors concluded that their screening tool was able to identify people living with moderate or severe disabilities but not very good at identifying people with minor disabilities.

In a qualitative study conducted in South Africa, Schneider [11] examined the difference that a word can make when measuring disability, and he found that participants perceived “disability” as an unchangeable and permanent state associated with physical disability and the inability to do anything. He concluded that the word “difficulty” should be used as it is in the WG tool instead of “disability”, for it constitutes a more comprehensive and inclusive measurement of disability. This recommendation to avoid the word “disability” has been supported by numerous studies [12, 13].

Schneider et al. [14] tested whether the WG short set of questions is appropriate for measuring disability in censuses in South Africa. Their results showed that the WG tool is a more inclusive measure of disability and provides clear information on severity. Indeed, the WG tool clearly screens the various aspects of disability (mental, physical and sensory) while taking into account the degree of severity. Mont [12] highlighted the necessity not only of using a scaled response instead of a yes/no response but also of presenting respondents with a range of prevalence. He showed that in Ecuador, for example, the prevalence of overall disability was 12.1% but was only 4% for severe disability. As with Schneider et al. [14], many other authors (such as Bachani et al. [15] and Palmer and Harley [8]) have concluded that—even if it is somewhat inconvenient [8, 13]—the WG screening instrument is highly relevant in surveys that collect self-reported information on general populations.

Schneider et al. [14] also analysed the trend of association between disability (as measured by the WG tool) and sociodemographic characteristics. Similarly to UNSD [16] and Bajekal et al. [17], they found that disability as measured by WG questions is positively associated with age. They also found that having functional difficulties is negatively associated with socioeconomic status (SES), education, employment and being in a union. Similar results were found by the South African statistical office in 2005 [18]. As did Schneider et al. [14], Mitra et al. [19] used an instrument very similar to that of WG, and they found that in most of the 16 developing countries of their sample, disability is positively associated with multidimensional poverty, lower education and unemployment.

To assess disability among adults aged 18 and above in a rural area of the Northwest Region of Cameroon, the International Centre for Evidence in Disability (ICED) [5] used both the WG extended question set and a clinical screening tool for vision, hearing, musculoskeletal impairment, epilepsy and clinical depression. In comparing the WG screening with the clinical screening, they found that the two instruments do not capture the same population: 46% of people who were clinically screened as disabled did not self-report functional limitation when the WG questions were used; whereas 22% of those who self-reported limitation with the WG tools was not clinically screened as having a disability. They found that the overall prevalence of disability among adults aged 18–49 was 6.9%. This prevalence was 5.1% when only the clinical screenings were used and 3.9% when only the WG extended set of questions were used. The authors found that people tend to self-report mostly severe clinical impairment and physical impairment. The study found no significant association between disability and gender. However, disability was positively associated with age and reports of serious health problems while it was negatively associated with having been in employment the week before the survey and having ever been married. Disabled adults were also more likely to be in the poorest quart.

In Cameroon, three nationally representative surveys collected information to measure adult disability: the Third Population and Housing Census of 2005 (3RGPH), the Cameroonian Household Survey (ECAM3), conducted in 2007, and the Demographic Health and Multiple Indicator Cluster Survey (DHS-MICS) of 2011. The screening instruments used in these surveys for measuring disability are very different from each other and from the one proposed by the WG. Additionally, two local studies specifically related to disability used the WG screening for disability assessment: the Disability and HIV study among adults in Yaoundé (HandiVIH) in 2015, and the 2014 North West Cameroon Disability Study (NWCDS), which was conducted among all ages of the population in a rural area located in the Northwest Region of the country. However, the latter survey is not publicly available and hence will not be used for the analysis presented below.

In this study, we aim to compare the disability measures provided by these three nationally representative datasets and to do so by using an external standard, namely the WG definition of disability used in the HandiVIH. More specifically, the following questions are addressed: What does each of these surveys measure as being a disability? Are there actually any differences in the adult disability prevalence that they measure? How do the different surveys observe the differentials among age, sex, education and socioeconomic groups? Are these differentials consistent across the surveys?

Answering these questions is very important. In fact, although these surveys contain great quantities of socioeconomic and demographic information, it is hard to say how accurate and comparable they are in terms of the results that could be obtained from crossing this information with disability. In fact, several studies have assessed the validity of the different disability measurements and most of them agree that the WG screening is the most relevant [8, 15, 20]. However, none of these studies has evaluated whether the associations between sociodemographic characteristics and disability measured by these instruments are comparable in a specific context. Is the association of sociodemographic characteristics with disability consistent whatever the screening instrument used? If yes, it would mean that these instruments capture the same disabled group profile but merely at different magnitudes. If not, it would mean that the use of some screening instruments produces more biased results among certain sociodemographic groups. This is particularly important in the case of Cameroon, where no nationally representative study has used the WG questionnaire and where the most recent study measuring disability uses a screening instrument that, to the best of our knowledge, has ever been assessed in the literature. We also go beyond the existing literature by assessing the comparability by type of disability.

## Methods

### Data

This study used four datasets. The first was the Third Population and Housing Census (3RGPH) dataset. This census was conducted in November 2005 by the Central Bureau of the Census and Population Studies (BUCREP) of the state of Cameroon with the aim of updating the sociodemographic characteristics of the population. In this study, we use the 10% sample of this census (1,772,359 individuals) provided by Integrated Public Use Microdata Series (IPUMS) International [21].

The second dataset that we used is the third Cameroonian Household survey (ECAM3), produced in 2007 by the Cameroon National Institute of Statistics (INS). The purpose of this survey was to update the economic conditions of the population and to evaluate the impact of major policies and programmes implemented to fight against poverty [22]. The survey was representative of the national population and targeted ordinary households.^1^ A total of 11,534 households were surveyed, including 51,232 individuals.

Third, we used the Demographic Health and Multiple Indicator Cluster Survey (DHS-MICS) of 2011. The survey was conducted by INS and the Ministry of Public Health with the support of ICF International, Centre Pasteur du Cameroun, the United Nations Population Fund, the United Nations International Children’s Emergency Fund, the US Agency for International Development and the World Bank. The purpose of the survey was to collect critical information on the health, socioeconomic and demographic characteristics of the population. A total of 14,214 households were surveyed but only half of them were administered the disability module through the household questionnaire [23]. Only this subsample of 35,177 individuals is analysed in this work.

Finally, we used the Disability and HIV study (HandiVIH) data as a “gold standard” for our comparison purpose. HandiVIH is a study conducted by the French Research Institute for Sustainable Development (IRD) and the Cameroonian Institute for Demographic Research and Training (IFORD) in the city of Yaoundé between October 2014 and November 2015. The aim of this study was to provide quantitative and qualitative insight into the complex links between disability and HIV in order to help decision-makers prioritise their interventions [24]. HandiVIH used a two-phase random sampling method. During the first phase, people were screened for disability from the general population using the WG disability questionnaire. Some basic socioeconomic information was also collected. In the second phase, their eligibility was confirmed for the interview. Only the screening dataset is used in our paper. One hundred seventy-seven enumeration areas in the town of Yaoundé were drawn from the national sampling framework that was provided by BUCREP, from each of which 200 households were randomly selected and all adults ages 15–49 were screened for disability. The sample was representative of the population of Yaoundé. Because HandiVIH is limited to adults aged 15–49, all the analyses of this paper are done for this age group only.

### Disability definitions and measures in Cameroonian surveys

The definition used in Cameroon by the Protection and Promotion of the Disabled Act is inspired by the ICF. According to this law, disability is “a limitation of opportunities for full participation of a person with impairment in an activity in a given environment” [25]. Although the ICF definition of disability is accepted worldwide, the instruments used to measure disability remain varied.

Table 4 in the Appendix 1 summarises the different instruments used by the different surveys in Cameroon to measure disability.

- The 3RGPH screened disability by asking the following direct question: “Has the person any chronic disease or any predominant handicap?” If yes, respondents reported the type(s) of disability from the 3RGPH list of disabilities (deaf, dumb, blind, leper, crippled upper limbs, crippled lower limbs, mental illness and albino).
- A similar instrument was used by the ECAM3, but with some variation. The question used was “Is (Name) the victim of a handicap?” If yes, what is the main handicap? The disability type options were visual, speech, hearing, mental and motor disability, deaf-dumb and other (to be specified). This screening method received a lot of criticism in the literature [26]. It captured mostly impairments of body structure/function [8]. Disability screening was not scaled by severity in these two surveys.
- DHS-MICS used the seven questions reported in Table 4 in the Appendix 1 to assess disability. It captures mostly body structure/function problems and considers the activity limitation component as well. Different functions are assessed: vision, hearing, mobility and cognitive functions. It also asks for reporting on whether these impairments or activity limitations are partial or total. For example, with respect to a deformation, there are two questions: (1) “Is there in your household someone suffering a deformation of the upper or lower limbs who cannot or finds it difficult to walk and/or use their arms or hands?” and (2) “Does (NAME) only have difficulty using his/her arms or legs, or can (NAME) not use his/her arms or legs at all?” The questionnaire also includes questions about the origin of the impairment. The DHS programme does not provide any recommendation or standard tool for disability measurement. The instrument used in the Cameroonian DHS-MICS was also used in the Chadian DHS of 2014, but it is otherwise not widely used and has received no validation test to the best of our knowledge.
- Even though HandiVIH is not representative at the national level, this survey has the advantage of having used the widely accepted WG instrument for screening disability. During the first stage of the interview, individuals were assessed for disability using six questions from the short set of the WG disability questionnaire and two additional questions from the extended set to better assess mental and intellectual disability [24]. Six functional domains were assessed: seeing, hearing, walking, cognition, self-care and communication. The WG questions capture mostly limitations in basic actions or domains [5, 20]. For example, sight is assessed using the following question “Do you have difficulty seeing even if wearing glasses?” The questionnaire also allowed for scaling the disability. For each function or action, people had to report if they experience no difficulty, some difficulties, a lot of difficulties or they are unable do it at all. The disability prevalence obtained using this instrument can be quiet different, depending on how this set of questions is used and the chosen severity cut-off [20].

The questionnaires for all these surveys were in French and English, the two official languages of the country. For 3RGPH, ECAM3 and DHS-MICS interviewers could also translate questions in local languages if interviewees were not comfortable with the official languages.

### Empirical strategy

In order to assess differences between prevalence estimates produced by a range of instruments, we followed the line of Hayes et al. [27] and calculated disability prevalence from each survey as well as their 95% confidence intervals (CI). Given that no clinical diagnosis has been done in Cameroon to assess the instruments used by the nationally representative survey, we use the WG instrument as our standard. Consequently, the disability prevalence was computed for Yaoundé, which is the region where the WG screening has been used and can be compared. Detailed analyses were conducted according to the age range, education, socioeconomic status and educational level of the respondent in order to evaluate the ability of these instruments to screen disability across the different groups. In addition, chi-squared tests were performed to assess the association of disability with these characteristics. For these analyses, we used the weights to ensure that samples reflect the population being analysed. No weight is available for HandiVIH, for which representativeness is ensured through the household sample selection.

In a second step, we used logistic regression models separately for each survey in the Yaoundé region to assess whether the associations between characteristics and disability were significant and if they were consistent across the surveys when controlling for the other characteristics. The odds ratios were calculated and reported.

Different disability variables are used in the study. First, we consider a dummy of overall disability status that takes the value 1 if the individual is disabled and 0 if not. For HandiVIH, we followed Beaudrap et al. [24] and Madans et al. [20], as our study identified disabled people as persons who reported either severe difficulties (a lot of difficulties or cannot perform at all) in at least one domain of the WG screening, or some difficulties in at least two of its domains. A dummy for the severity of disability is also constructed for DHS-MICS and HandiVIH. We considered as severely disabled any person who reported in the DHS-MICS that his/her disability is total and in HandiVIH that they are unable to do any action from the WG questions.

In order to account for the different aspects of the disability, we consider the three variables of motor, sensory and mental disability. This is in alignment with the distinctions made by the Cameroonian law on disability [25].^2^ The variable for *motor disability* takes the value 1 if the person has physical impairments or mobility limitation and 0 if not. The *sensory disability* variable indicates whether or not the person was reported as having any hearing, visual or speaking impairment. The *mental disability* variable takes the value 1 if the person has a behavioural, intellectual or mental disorder and 0 if not. Table 5 in the Appendix 2 reports more details about the grouping of conditions for each survey. HandiVIH is similar to the overall measurement of disability in that a person is considered to have a specific type of impairment either if (s)he has severe difficulties in at least one domain reported in the corresponding cell of Table 5 in the Appendix 2 or if (s)he has some difficulties in at least 2 domains reported in the cell.

The variables age group, education, marital status and tercile of socioeconomic status (SES) are used as dependent variables to assess their associations with disability. The age group variable is a three-age-group categorical variable (15–24, 25–34 and 35–49 age groups). Education is a dummy variable taking the value 0 if the individual has no education or did not complete primary school and 1 if (s)he completed primary school or studied more years. The marital status indicates whether the individual is in a union or not. The SES for the 3RGPH and for HandiVIH surveys has been calculated from household assets using the principal component analysis method [28]. That variable was provided with the DHS-MICS dataset and was calculated by the data producers using a similar methodology to that of the 3RGPH and HandiVIH [23]. The well-being measurement in the ECAM3 data is an index of consumption by head calculated by INS as an equivalent to the household consumption aggregate per adult [22].

## Results

### Comparison of disability prevalence and association across the surveys

As we can observe from the first panel of Table 1, the adult disability prevalence at the national level is significantly different from one survey to another. The highest prevalence comes from the DHS-MICS survey (4.7% CI = [4.3, 5.2]), followed by ECAM3 (3.5% CI [3.2, 3.6]) and then RGPH (1.5%, CI = [1.4, 1.5]). The prevalence rates of different types of disabilities are also different from one survey to another, with the highest prevalence rates of motor, sensorial and mental disabilities found when using the DHS-MICS instrument.

**Table 1:** Prevalence of the type of disability in each survey (15–49 age group)

	3RGPH 2005		ECAM3 2007		DHS-MICS 2011		HandiVIH 2015	
	%	95% CI	%	95% CI	%	95% CI	%	95% CI
Country level								
Motor disability	0.29	[0.28,0.30]	1.19	[1.03, 1.38]	1.71	[1.47, 1.99]	--	
Sensory disability	0.78	[0.76, 0.80]	1.67	[1.48, 1.87]	2.61	[2.31, 2.96]	--	
Mental disability	0.09	[0.08, 0.10]	0.40	[0.32, 0.50]	0.77	[0.63, 0.93]	--	
Other disability	0.30	[0.29, 0.31]	0.22	[0.15, 0.32]	--		--	
Any disability	1.46	[1.43, 1.49]	3.48	[3.20, 3.78]	4.74	[4.32, 5.20]	--	
Severe disability	--		--		0.71	[0.57, 0.88]	--	
Observations	820797		24830		15011		--	
Yaoundé								
Motor disability	0.26	[0.23, 0.29]	0.77	[0.482, 1.21]	1.14	[0.71, 1.81]	0.82	[0.74, 0.90]
Sensory disability	0.55	[0.51, 0.60]	1.67	[1.21, 2.31]	2.18	[1.00, 3.24]	0.82	[0.74, 0.90]
Mental disability	0.06	[0.05, 0.08]	0.21	[0.10, 0.45]	0.53	[0.25, 1.10]	0.55	[0.49, 0.62]
Other disability	0.18	[0.15, 0.20]	0.10	[0.04, 0.27]	--		0.25	[0.21, 0.29]
Any disability	1.04	[0.98, 1.10]	2.75	[2.15, 3.51]	3.62	[2.54, 5.14]	2.59	[2.45, 2.73]
Severe disability	--		--		0.39	[0.18, 0.82]	0.29	[0.24, 0.34]
Observations	108579		2474		1443		49038	

The second panel of Table 1 compares the prevalence of disability in these Yaoundé city surveys with that obtained from HandiVIH, which serves as a reference. Once again, the highest prevalence is found with the DHS-MICS instrument (3.6% CI = [2.5, 5.1]), followed by ECAM3 (2.7% CI = [2.1, 3.5]), HandiVIH (2.6 [2.4, 2.7]) and finally 3RGPH (1.0% [1.0, 1.1]). However, we can see that the CI of ECAM3 and DHS-MICS overlap with that of HandiVIH. Hence, the prevalence rates of disability found using the disability screening instrument of these surveys are not significantly different from those found using the WG short set questionnaire. The prevalence obtained when using the 3RGPH tools is significantly lower than the one obtained by the WG questions.

When the DHS-MICS survey refers to the severity of disability as a non-partial disability, this seems to be very similar to what is measured by the WG tools when they define the severity of disability. In fact, 0.4% (CI = [0.2, 0.8]) were identified with non-partial disability in DHS-MICS while 0.3% (CI = [0.2, 0.3]) of people reported being unable to do any one of the 6 actions addressed in HandiVIH.

Similarly, when we consider the types of disabilities in Yaoundé, we notice that the prevalence rates of motor and mental disabilities obtained through DHS-MICS (1.1% CI = [0.7, 1.8] and 0.5% CI = [0.2, 1.1], respectively) are not significantly different from those obtained through HandiVIH (0.8% CI = [0.7, 0.9] and 0.5% CI = [0.5, 0.6], respectively). But there is a difference in the sensory disability prevalence rates (2.2% CI = [1.0, 3.7] in DHS-MCS and 0.8% CI = [0.7, 0.9] in HandiVIH). Although the prevalence of motor disability obtained from ECAM3 (0.8% CI = [0.5, 1.2]) does not differ significantly from that of HandiVIH, the prevalence of sensory disability is significantly higher (1.8% CI = [1.2, 2.3]), while that of mental disability is significantly lower (0.2% CI = [0.1, 0.4]). Whatever the nature of the disability, prevalence rates obtained from 3RGPH remain significantly lower (0.3% CI = [0.2, 0.3], 0.5% CI = [0.5, 0.6], 0.1% CI = [0.0, 0.1], respectively).

Table 2 presents the bivariate analyses of the association between sociodemographic characteristics and disability in Yaoundé. The *p* values of the chi-squared test show that disability is significantly associated with education in all the surveys. In fact, as educational level increases, disability prevalence tends to be lower in all the surveys, going from 1.9% to 0.9%, 5.0% to 2.4%, 8.4% to 3.1% and 7.6% to 2.1% in, respectively, 3RGPH, ECAM3, DHS-MICS and HandiVIH.

**Table 2:** Disability prevalence by demographic and socioeconomic characteristics in each survey (15–49 age group)

	3RGPH		ECAM3		DHS-MICS		DHS-MICS severe disability		HANDIVIH		HANDIVIH severe disability	
	Row%	95%CI	Row%	95%CI	Row%	95%CI	ROW%	95%CI	Row%	95%CI	Row%	95%CI
Age group												
15–24	0.9	[0.8, 0.9]	2.3	[1.5, 3.4]	3.9	[2.3, 6.5]	0.3	[0.1, 1.4]	2.0	[1.8, 2.2]	0.2	[0.2, 0.3]
25–34	1.1	[1.0, 1.2]	2.5	[1.7, 3.8]	3.2	[1.9, 5.2]	0.6	[0.2, 1.6]	2.3	[2.1, 2.5]	0.2	[0.2, 0.3]
35–49	1.3	[1.2, 1.5]	4.0	[2.6, 6.2]	3.8	[2.0, 7.0]	0.2	[0.0, 1.7]	4.0	[3.7, 4.4]	0.5	[0.4, 0.6]
		*P* = 0.0000		*P* = 0.130		*P* = 0.807		*P* = 0.612		*P* = 0.000		*P* = 0.000
Education												
None	1.9	[1.6, 2.3]	5.0	[2.8, 8.9]	8.4	[4.4, 15.4]	2.5	[0.8, 7.2]	7.6	[6.9, 8.5]	1.5	[1.2, 1.9]
Primary or more	0.9	[0.9, 1.0]	2.4	[1.8, 3.2]	3.1	[2.1, 4.6]	0.2	[0.1, 0.5]	2.1	[2.0, 2.3]	0.2	[0.1, 0.2]
		*P* = 0.000		*P* = 0.023		*P* = 0.003		*P* = 0.000		*P* = 0.000		*P* = 0.000
SES												
Poorest	1.1	[1.0, 1.2]	3.9	[2.6, 5.7]	5.2	[3.4, 7.9]	0.7	[0.3, 2.0]	3.1	[2.9, 3.4]	0.4	[0.3, 0.5]
Middle	1.0	[0.9, 1.1]	2.1	[1.2, 3.4]	3.0	[1.5, 6.0]	0.3	[0.1, 1.2]	2.2	[2.0, 2.4]	0.2	[0.2, 0.3]
Richest	1.0	[0.9, 1.1]	2.5	[1.7, 3.7]	2.7	[1.2, 6.1]	0.2	[0.0, 1.3]	2.2	[2.0, 2.5]	0.2	[0.1, 0.3]
		*P* = 0.129		*P* = 0.093		*P* = 0.242		*P* = 0.259		*P* = 0.000		*P* = 0.004
Sex												
Female	0.9	[0.9, 1.0]	1.4	[0.7, 2.7]	3.5	[2.1, 5.7]	0.2	[0.1, 0.9]	2.6	[2.5, 2.8]	0.2	[0.2, 0.3]
Male	1.1	[1.0, 1.2]	3.2	[2.4, 4.1]	3.8	[2.4, 5.9]	0.6	[0.2, 1.4]	2.5	[2.3, 2.7]	0.4	[0.3, 0.5]
		*P* = 0.002		*P* = 0.019		*P* = 0.816		*P* = 0.249		*P* = 0.428		*P* = 0.000
Union status												
Not in union	1.1	[1.0, 1.2]	2.8	[2.1, 3.8]	3.9	[2.5, 6.0]	0.6	[0.3, 1.4]	3.0	[2.8, 3.2]	0.4	[0.3, 0.5]
In union	1.0	[0.9, 1.1]	2.6	[1.6, 4.0]	3.4	[1.9, 5.8]	0.1	[0.0, 0.7]	2.1	[1.9, 2.3]	0.2	[0.1, 0.2]
		*P* = 0.075		*P* = 0.734		*P* = 0.684		*P* = 0.056		*P* = 0.000		*P* = 0.000
TOTAL	1.0	[1.0, 1.1]	2.7	[2.2, 3.5]	3.6	[2.6, 5.2]	0.4	[0.2, 0.8]	2.6	[2.5, 2.7]	0.3	[0.2, 0.3]

Authors calculations from 3RGPPH, ECAM3, DHS-MICS and HandiVIH. Pr are *p* values of the chi-squared test

Results show that, whatever the screening instrument, there is in general a positive association between age and disability, though this association is significant only for HandiVIH and 3RGPH. There is a significant association (at the 10% level) between disability and SES only in HandiVIH and ECAM3. In these surveys, the prevalence of disability is higher among the poorest (3.1% and 3.9%, respectively). A negative association between disability and being in a union is found only in HandiVIH and 3RGPH. Finally, there is no association between sex and disability in DHS-MICS and HandiVIH, while in ECAM3 and 3RGPH, disability is positively and significantly associated with being a male.

Concerning severe disability, using the HandiVIH tool finds that it is positively associated with age and being male while it is negatively associated with education, SES and being in a union. In DHS-MICS, severe disability is found to be significantly and negatively associated with education and union status only. However, all these association assessments must be considered more deeply through multivariate analyses.

### Multivariate analyses

After controlling for the other factors, the assessed association of disability with each sociodemographic factor is presented in Table 3. The results show that, whatever the instrument used, there is a significant and negative association between disability and education. Compared to adults with no education, and controlling for other characteristics, the odds of being disabled for those with any education is 50%, 40%, 70% and 70% lower in, respectively, 3RGPH, ECAM3, DHS-MICS and HandiVIH. Similarly, the odds of being disabled is lower for individuals with better SES in all the surveys when all the disabilities are considered; however, this association disappears when only severe disabilities are considered in HandiVIH and DHS-MICS surveys.

**Table 3:** Odds ratio from logistic regression of disability on sociodemographic characteristics for the Yaoundé region (15–49 age group)

	3RGPH		ECAM3		DHS-MICS		DHS-MICS Severe disability		HandiVIH		HandiVIH Severe disability	
	OR	95%CI	OR	95%CI	OR	95%CI	OR	95%CI	OR	95%CI	OR	95%CI
Age group [15–24]												
25–34	1.4***	[1.2, 1.7]	1.7*	[0.9, 2.9]	1.1	[0.5, 2.1]	8.8**	[1.2, 62.1]	1.5***	[1.3, 1.8]	1.3	[0.9, 2.0]
35–49	2.0***	[1.7, 2.3]	2.8***	[1.5, 5.4]	1.4	[0.6, 3.0]	3.4	[0.2, 48.5]	3.1***	[2.7, 3.6]	3.6***	[2.3, 5.5]
Education												
[None]												
Primary or more	0.5***	[0.4, 0.6]	0.6*	[0.3, 1.1]	0.3***	[0.2, 0.7]	0.1***	[0.0, 0.3]	0.3***	[0.2, 0.3]	0.1***	[0.1, 0.1]
SES												
[Poorest]												
Middle	0.9*	[0.7, 1.0]	0.5*	[0.3, 1.0]	0.5*	[0.3, 1.0]	0.4	[0.1, 2.3]	0.8***	[0.7, 0.9]	0.8	[0.6, 1.2]
Richest	0.9	[0.8, 1.1]	0.7	[0.4, 1.2]	0.5*	[0.3, 1.1]	0.2	[0.0, 1.9]	0.8**	[0.7, 1.0]	0.8	[0.5, 1.4]
Male	1.2**	[1.0, 1.3]	2.1**	[1.1, 4.2]	1.1	[0.6, 1.8]	3.7	[0.6, 21.3]	0.9	[0.8, 1.1]	1.9***	[1.4, 2.7]
In union	0.7***	[0.6, 0.8]	0.6*	[0.3, 1.0]	0.8	[0.4, 1.5]	0.1**	[0.0, 0.7]	0.4***	[0.4, 0.5]	0.2***	[0.1, 0.3]
Observations	105852		2425		1423		1423		48900		48900	

Significance levels: *p < 0.1, **p < 0.05, ***p < 0.01. Variables in parentheses are reference categories

*OR*, odds ratio; *CI*, confidence interval

In all the surveys except DHS-MICS, having any disability is positively associated with age and negatively associated with being in a union. There is no significant association in DHS-MICS between any disability and age and union status even if the coefficients show the same direction of association that is found for the other surveys. However, when severe disability is considered, we can see that in DHS-MICS the odds of being disabled for adults aged 25–49 is 8.8 times the odds of adults aged 15–24. The odds of being severely disabled are 90% lower for those in a union compared to those who are single.

Controlling for all the other characteristics, the odds of reporting any disability is higher for males compared to females only when the 3RGPH and ECAM3 instruments are used. For DHS-MICS and HandiVIH, no association is found between sexes and reporting any disability. Yet the odds of reporting severe disability in HandiVIH is significantly higher for males (OR = 1.9) compared to women.

## Discussion

The aim of this study was to assess the comparability of the disability measurements used in Cameroonian nationally representative surveys and censuses, using the HandiVIH local survey as external reference. More specifically, it highlighted what each of these surveys measures as disability and then checked whether the prevalence rates of disability measured by them are significantly different. Finally, it assessed the association of disability with sociodemographic characteristics in each of these surveys and evaluated their consistency across surveys. Different results have been found.

We highlighted that 3RGPH and ECAM3 disability measurement tools capture mostly body function and structural problems/impairments through a direct question using the word “handicap”. The DHS-MICS, for its part, captures body function and structural limitations, but also some activity limitations. It also captures severity by asking whether the disability is partial or not. HandiVIH measures activity limitation through the WG short set of questions and two additional questions from the extended set on cognitive limitations. The severity of the disability is also scaled, thus allowing the implementation of different severity cut-off scores.

Calculating the proportion of disabled people in the city of Yaoundé shows that, overall, the disability prevalence rates obtained by the ECAM3 and DHS-MICS instruments are not significantly different from those obtained using the WG screening tool in HandiVIH. On the other hand, the prevalence of disability when the 3RGPH screening is used is significantly lower than in HandiVIH. This confirms the results of Schneider et al. [14], who found that the prevalence obtained with the direct question using the term “disability” was lower in South Africa.

It is also important to note that the screening instruments of ECAM3 and RGPH—which at first seem similar—produce very different prevalence rates. Both use the word “handicap”, but in different ways. First, the lists of disabilities provided as examples in each of these surveys are different, with 3RGPH somehow having more severe conditions (see Table 5 in the Appendix 2). Second, the RGPH uses the question “Has the person any chronic disease or any predominant handicap?” while ECAM3 uses the question “Is (Name) the victim of a handicap?” Thus, the wording seems to matter, as using the word “victim” instead of “have” seems to make people more comfortable in reporting their disabilities.

When we look at the prevalence by type of disability, we find that the prevalence of disability in 3RGPH is the lowest regardless of whether the disability is motor, sensorial or mental, thereby confirming that only very severe and visible forms of disability are reported in this survey. The code list given to the interviewers confirms this assumption, as the reported disability is presumed to fall under functional impairment (deaf, mute, blind, crippled upper and lower limbs, mental disease), leprosy or albinism.

Despite the fact that using the word “disability” is supposed to capture only severe forms of disability, the relatively high prevalence of disability in ECAM3 is due to sensorial disability, which is significantly higher than even in HandiVIH. The instrument used in ECAM3 does not take into account the possible compensation that can be provided by devices such as eyeglasses or hearing aids for the two most frequent sensorial limitations (difficulty in seeing and hearing). By contrast, the WG tool used in HandiVIH is very different. Persons with compensated limitations (e.g. having no difficulty in seeing when wearing eyeglasses) are not considered to be disabled. In parallel, mental disability prevalence in ECAM3 is significantly lower than in HandiVIH. When looking at the different examples given to the interviewers for mental disability in ECAM3, it refers only to very stigmatised mental diseases (“insane, alienated, crazy, etc.”), whereas HandiVIH refers to cognitive issues (remembering, concentrating, learning, analysing).

As with ECAM3, the instrument used in DHS-MICS for reporting sensorial limitations does not take into account any possible compensation, which leads to a higher prevalence of sensorial disability than in HandiVIH. For motor limitations, DHS-MICS reports both people with impairments (even those suffering no consequences in their daily lives, such as a person missing a toe) and people with restrictions in their activities (e.g. difficulty in walking), whereas HandiVIH identifies only the latter. For mental disability, DHS-MICS and HandiVIH report exactly the same prevalence; however, DHS-MICS reports people with “behavioural disorders” in general while HandiVIH refers to cognitive issues in the manner already mentioned.

These results show that different formulations of any direct question that uses the word “handicap” for screening disability will ultimately not produce comparable results. This corroborates the findings of Schneider [11], namely that—as far as disability measures are concerned—the words used are very important, even though his work was limited to the difference between using “difficulty” and “disability”. Whether they are given as examples or as guidelines for interviewers, the possible answers to expect from such questions also highlight the different expectations of exactly what the survey’s designers intend to find with this question. Our results confirm that researchers should exercise extreme caution when comparing surveys used to analyse the prevalence of disability, as they each use different tools.

It is worth mentioning that because Cameroon has two official languages, French and English questionnaires were available for each of the four surveys and census. The language used was the one the interviewee felt more comfortable with. In addition, interviewers were allowed to translate the questions in local languages if the respondents were comfortable with none of the two official languages. The language used could impact the reporting of disability. DHS-MICS was the only survey to provide such information in the database and we found no statistical association between disability and the language of the questionnaire.^3^ Translation in local language was rarely done in Yaounde (six observations), which is an urban area: hence, we could not control for this information.

Even though the screening tool of DHS-MICS has never received any formal validation in the literature, our comparison seems to indicate that it produces prevalence rates in Yaoundé that are not significantly different from those obtained from the WG instrument employed in HandiVIH. However, despite the similar prevalence levels, the identified groups are not necessarily the same between the two surveys.

Comparing disability rates by looking at the different types of disability has some limitations. The approach that consists of looking at the degree of severity appears to be a good alternative solution whenever it is possible to assess this. As already mentioned, 3RGPH reports only very severe forms, while DHS-MICS and HandiVIH offer the possibility of estimating severe disability; unfortunately, neither of these is possible with ECAM3, which indicates disability prevalence by combining severe forms of motor and mental disabilities with more moderate forms of sensorial disability.

In order to go beyond the simple comparison of the prevalence rates, we proposed a multivariate analysis that can assess the demographic and socioeconomic similitude of the disabled persons identified across the surveys.

Regarding the association of disability with sociodemographic characteristics, logistic regressions show that—for HandiVIH overall—using the WG screening tool as a measure finds that disability is positively associated with age while it is negatively associated with educational level, SES and being in union and it is not associated with sex. The results for education, union status, SES and sex are similar to what has been found by ICED in the Northwest Region [5].

When looking at severe disability screened by the WG tool, SES is not associated with disability to any greater degree, and males tend to be two times more disabled than females. The same result is found for SES in DHS-MICS—a negative association with disability and no association with severe forms only—while 3RGPH does not show a strong association between SES and disability. This contradicts what has been found in the literature [19]. However, some studies have arrived at different figures [29]. In the context of Yaoundé, we can hypothesise that, contrary to more moderate forms, severe forms of disability are difficult to compensate for, even among the richest people.

With the exception of DHS-MICS, there is also an important distinction between males and females. Severe forms of disability reported in HandiVIH and in 3RGPH appear to be more frequent among males than females, and various explanations can be put forward for these different disability patterns between the sexes. For example, the different behaviours of women and men result in them suffering different diseases in high-income countries [30]. Also, in Yaoundé, men are presumed to be exposed to more severe disabling diseases or injuries than women as a result of their daily life behaviours (professional activity, driving, diet, alcohol consumption, etc.). In addition, the literature shows that men tend to have more complex situations due to their having a combination of disabilities that are more likely to generate activity restrictions [31]. To date, little is known about gender differences in disability in Africa, although recent studies have explored this issue and delivered mixed figures, depending on the context [32–34].

Meanwhile, education remains strongly significant whatever the survey, as found in the literature [19]. On the one hand, disability that has existed since birth or childhood has probably decreased the probability of going to school [35]; for adults, on the other hand, the professional and/or daily life activities of people who are not educated can lead to greater probabilities of having a disability than would be the case with educated persons [36]. As for education, not being in a union is strongly associated with disability, especially when the disability is severe: on the one hand, being disabled decreases the probability of entering into a union [37] while, on the other, becoming disabled can weaken the union [38]. Finally, as expected, age is positively associated with disability except in DHS-MICS. In DHS-MICS, for severe disability, the risk for the oldest is similar to that of the youngest. However, compared to the youngest, the middle age group is more likely to be severely disabled.

This study nevertheless has some limitations. First, it compares surveys conducted at different points in time. Hence, the prevalence of disability may have changed between them, although we do not expect there to be any dramatic changes—especially between the 2005 and 2007 surveys on the one hand and between the 2011 and 2015 surveys on the other. Therefore, we think that most of the variations are attributable to the screening tool.

Second, the comparability of screening instruments has been done using different surveys conducted on different samples. This could limit the comparability of results. Nevertheless, all the surveys are nationally representative, except HandiVIH, and representative of the population of Yaounde, except ECAM3. Hence, if the comparability is not possible at the individual level, the prevalence at the national and at the level of Yaounde are comparable for most of the surveys and the differences found could be attributable to the instruments used.

Third, the HandiVIH survey that we use as our reference/standard was conducted only in Yaoundé, thus our comparison of disability measurement concerns solely this city. Our results on multivariate analysis may not be valid for other areas of the country, though they are very similar to what ICED found for the Northwest Region [5]. Forth, being restricted to the city of Yaoundé leaves us with a small sample of disabled people, especially from the DHS-MICS survey, and this could explain why most of the associations obtained using this survey are not statistically significant. Finally, we had to choose a severity threshold to define the disability when using the WG tool, and the change to this threshold may have modified the prevalence obtained^4^.

## Conclusion

This study contributes to the literature in different ways. First, it is the first time any study has evaluated the comparability of a nationally representative survey with a survey using the WG screening tool in Cameroon, a country which has never run a national representative survey using the internationally recognised WG disability screening tool. Second, it is the first time a study has ever compared the association of sociodemographic characteristics with disability measures using different instruments in a specific context. This allows assessing if the association trends are similar to what is observed with WG, meaning either that the response bias using non-conventional screening tools is homogenously distributed among the groups or—if they are divergent—that some groups are more or less likely to report disability depending on the screening tool used. The analyses have been conducted according to type of disability in order to obtain more precise results. Finally, the study provides an important set of empirical recommendations.

None of the instruments used in the Cameroon nationally representative surveys produced both disability prevalence and association trends that were exactly similar to those obtained using the WG disability screening tool. Although ECAM3 and 3RGPH both use the word “handicap” in their screening questions, they produce significantly different results, which serves as a warning against consideration of any study that takes it for granted that they are similar. The DHS-MICS instrument, which until now has never been tested, allows obtaining motor, mental and severe disability prevalence rates that are, overall, not significantly different from those obtained using the WG. Thus, this measure can be judged as trustworthy for that purpose. However, given that there is no standard instrument in Demographic and Health Surveys across countries, the WG screening standard continues to be pertinent for the international comparability of results from studies on disability.

## Data Availability

The 3RGPH data that were analysed during the current study are available in the IPUMS international repository at https://international.ipums.org/international/index.shtml. The DHS-MICS data are available in the DHS Programme repository at https://dhsprogram.com/what-we-do/survey-Types/dHs.cfm. The ECAM3 data are available from the INS upon reasonable request at http://www.statistics-cameroon.org/index.php. The HandiVIH data are available from the principal investigators Pierre Debeaudrap and Gervais Beninguisse upon reasonable request.

## Appendix 1

**Table 4:** Disability measurement in Cameroonian surveys

Survey	Date	Producer	Screening	Questions and disability characteristics	Type of disability	Functional domain	Dichotomous/scaled	Self-reported?	Age range	Representativeness
3RGPH	2005	BUCREP (10% IPUMS’s dataset)	Direct question using the word “disability”	“Has the person any chronic disease or any predominant handicap?” List of handicaps selected by 3RGPH: deaf, mute, blind, crippled upper and lower limbs, mental disease + leprosy + albinism	Physical, mental, sensory	Body structure/function	Yes/no	No (one referent person for all the household members or the interviewer if the disability was noticeable)	All ages	Cameroon
ECAM3	2007	INS	Direct question using the word “disability”	“Is (Name) victim of a handicap?” If yes, what is the main handicap? List of functional impairments (visual, speaking, auditory, mental and motor disability, deaf-mute, other)	Physical, mental, sensory	Body structure/function	Yes/no	No (one referent person for all the household members)	All ages	Cameroon
DHS-MICS	2011	Macro International	Other screening instrument	-“Is there a person in your household who is missing a body part, for example, a hand, arm, foot or leg?” -“Is there a person in your household who is missing an extremity, such as a fingertip, toe, nose or ear?” -“Is there a person in your household who has a deformed upper or lower limb and cannot, or only with difficulty, walk and/or use their arms or hands?” -“Is there a person in your household who can hardly see or is blind?” -“Is there a person in your household who can hardly hear or is deaf?”-“Is there a person in your household who finds it very hard to speak or is dumb?” -“Is there a person in your household who has behavioural disorders ”	Physical, mental, sensory	Body structure/function and some activity limitation	Yes/no and if yes: partial/total	No (one referent person for all the household members)	All ages	Cameroon
HandiVIH	2015	IFORD & IRD	WG short set screening for disability assessment +2 questions from the WG extended questionnaire for mental disability	-“Do you have difficulty seeing even if wearing glasses?” -“Do you have difficulty hearing even if using hearing aid/s or are you deaf?” -“Do you have difficulty walking or climbing stairs?” -“Do you have difficulty remembering or concentrating?” -“Do you have difficulty (with self-care, such as) washing all over or dressing?” -“Do you have difficulty communicating (for example, understanding or being understood by others)?” -“Do you have difficulty learning a new task, for example learning how to get to a new place?” -“Do you have difficulty analysing and finding solutions to problems in day-to-day life?”	Physical, mental, sensory	Body structure/function and activities (with self-care such as washing all over or dressing)	Scaled (No, Some, A lot, and Unable)	Yes	15–49	Yaoundé

## Appendix 2

**Table 5:** Grouping by type of impairment, according to the survey

Survey	Motor disability	Sensorial disability	Mental disability	Other disability
3RGPH	Crippled upper limbs, crippled lower limbs	Deaf, dumb, blinded	Mental illness,	Albino, leprosy
ECAM3	Crippled, paralytic	Blind, visually impaired, stutter, mute, deaf, hearing impaired, deaf-mute, etc.	Insane, alienated, crazy, etc.	Other, or missing value for disability type even though reported as disabled
DHS-MICS	Lack of a body part or “extremity”, deformation of an upper or lower limb, unable or has some difficulties walking or using arm	Blind, visually impaired, dumb or hearing impaired, mute or serious speaking impairments	Behavioural disorders	NA
HandiVIH	Difficulty walking or climbing stairs Difficulty with self-care	Difficulty seeing even if wearing glasses, Difficulty hearing even if using hearing aid/s or deaf	Difficulty remembering or concentrating Difficulty learning a new task, Difficulty analysing and finding solutions to problems	Difficulty communicating

## Abbreviations

BUCREP: Central Bureau of the Census and Population Studies
CI: Confidence intervals
DHS-MICS: Demographic Health-Multiple Indicator Cluster Survey
ECAM3: Cameroonian Household Survey
HandiVIH: A disability and HIV study among adults conducted in Yaoundé
ICED: International Centre for Evidence in Disability
ICF: International Classification of Functioning, Disability and Health
IFORD: Institut de Formation et de Recherche Démographiques
INS: Cameroon’s National Institute of Statistical
IRD: Institut de Recherche pour le Développement
LMIC: Low- and middle-income countries
NWCDS: North-West Cameroon Disability Study
Pp: Percentage point
3RGPH: Cameroonian Population and Housing Census
SES: Socioeconomic status
WG: Washington Group on Disability Statistics
WHO: World Health Organisation
WHS: World Health Surveys
UN: United Nations

## Footnotes

1 Ordinary household is the opposite of collective households which include boarding schools, barracks, hospitals, and convents.

2 For simplicity reasons, we did not make a separate analysis for poly-disabled, which is also one of the categories included in the law.

3 Results available upon request. Similar results are obtained when the language of the questionnaire is used as control in the multivariable analyses.

4 However, we tried different thresholds and adopted the one that is widely accepted in the literature and that provides results closest to those obtained with the other surveys.

## References

[1] Mbouyap Y-M, Ahanda JM. Situation Socio-Economique des Personnes vivant avec un Handicap. BUCREP; 2010. http://www.bucrep.cm/index.php/fr/component/phocadownload/category/42-analyses-thmatiques. Accessed 18 Jun 2015.

[2] Simo Fotso A, Zamo Akono CM, Tsafack Nanfosso R. Disability and labour force participation in Cameroon. In: Kayizzi-Mugerwa S, Shimeles A, Lusigi A, Moummi A, editors. Inclusive Growth in Africa: Policies, Practice, and Lessons Learnt. Routledge; 2016.

[3] INS. Enquête par Grappes à Indicateurs Multiples (MICS3), Deuxième série de MICS au Cameroun - Aperçu. Final report. Yaoundé, Cameroun: INS; 2006. http://slmp-550-104.slc.westdc.net/~stat54/nada/index.php/catalog/19/study-description. Accessed 14 Nov 2016.

[4] Cockburn L, Cleaver S, Benuh E. The prevalence of impairments and disabilities in the North West Region, Cameroon. Health Sci Dis. 2014;15. http://www.hsd-fmsb.org/index.php/hsd/article/view/332. Accessed 14 Nov 2016.

[5] ICED. The North West Cameroon Disability Study Country Report. , London School of Hygiene and Tropical Medicine (LSHTM}; 2014. http://disabilitycentre.lshtm.ac.uk/files/2014/12/Cameroon-Country-Report.pdf.

[6] WHO. International classification of functioning, disability and health: ICF. World Health Organization; 2001.

[7] Melanie K (2008). Jones. Disability and the labour market: a review of the empirical evidence. J Econ Stud..

[8] Palmer M, Harley D (2012). Models and measurement in disability: an international review. Health Policy Plan..

[9] Ingstad BI, Whyte SR. Disability and culture. University of California Press. London; 1995. https://www.ucpress.edu/book.php?isbn = 9780520083622. Accessed 9 Mar 2018.

[10] WHO. ICF Checklist Version 2.1a, Clinician form for international classification of functioning, disability and health. 2003. http://www.who.int/classifications/icf/training/icfchecklist.pdf.

[11] Schneider M (2009). The difference a word makes: responding to questions on “disability” and “difficulty” in South Africa. Disabil Rehabil..

[12] Mont D. Measuring disability prevalence. Discussion paper. The World Bank; 2007. http://documents.worldbank.org/curated/en/578731468323969519/Measuring-disability-prevalence. Accessed 8 Mar 2018.

[13] Swartz L. Five challenges for disability-related research in sub-Saharan Africa. Afr J Disabil. 2014;3. 10.4102/ajod.v3i2.149. 10.4102/ajod.v3i2.149 PMC5442506 28730014

[14] Schneider M, Dasappa P, Khan N, Khan A (2009). Measuring disability in censuses: the case of South Africa. ALTER - Eur J Disabil Res Rev Eur Rech Sur Handicap..

[15] Bachani AM, Galiwango E, Kadobera D, Bentley JA, Bishai D, Wegener S (2014). A new screening instrument for disability in low-income and middle-income settings: application at the Iganga-Mayuge Demographic Surveillance System (IM-DSS). Uganda. BMJ Open..

[16] UNSD. Disability Statistics Compendium. Statistics on Special Population Groups. New York: UNSD Department of International Economic and Social Affairs Statistical Office; 1990. https://unstats.un.org/unsd/publication/seriesy/seriesy_4e.pdf. Accessed 9 Mar 2018.

[17] Bajekal M, Harries T, Breman R, Woodfield K. Review of disability estimates and definitions. UK: Department for Work and Pensions; 2004. http://www.eurohex.eu/bibliography/pdf/Bajekal_reportDWP_2004-0697451521/Bajekal_reportDWP_2004.pdf. Accessed 9 Mar 2018.

[18] StatSA. Census 2001: prevalence of disability in South Africa. Pretoria; 2005. http://www.statssa.gov.za/census/census_2001/disability/Disability.pdf.

[19] Mitra S, Posarac A, Vick B (2013). Disability and poverty in developing countries: a multidimensional study. World Dev..

[20] Madans JH, Loeb ME, Altman BM (2011). Measuring disability and monitoring the UN Convention on the Rights of Persons with Disabilities: the work of the Washington Group on Disability Statistics. BMC Public Health..

[21] Minnesota Population Center. Integrated public use microdata series, International: Version 6.4 Cameroon Third Population and Housing Census. Data. Minneapolis, MN: University of Minnesota: IPUMS-International; 2015. 10.18128/D020.V7.0*.*

[22] INS. Troisième Enquête Camerounaise auprès des Ménages. Final report. INS; 2008. http://nada.stat.cm/index.php/catalog/18. Accessed 14 Nov 2016.

[23] INS. Enquête Démographique et de Santé et à Indicateurs Multiples. Rapport final. INS; 2012.

[24] De Beaudrap P, Pasquier E, Tchoumkeu A, Touko A, Essomba F, Brus A (2016). HandiVIH—A population-based survey to understand the vulnerability of people with disabilities to HIV and other sexual and reproductive health problems in Cameroon: protocol and methodological considerations. BMJ Open..

[25] Cameroun. Loi No 2010/002 du 13 Avril 2010 Portant sur la Protection et la Promotion des Personnes Handicapées. 2010. http://www.mindbank.info/item/2312. Accessed 19 Jun 2015.

[26] Schneider Marguerite (2016). Cross-National Issues in Disability Data Collection. International Measurement of Disability.

[27] Hayes RD, Dennerstein L, Bennett CM, Fairley CK (2008). What is the “true” prevalence of female sexual dysfunctions and does the way we assess these conditions have an impact?. J Sex Med..

[28] Filmer D, Pritchett LH (2001). Estimating wealth effects without expenditure data--or tears: an application to educational enrollments in states of India. Demography..

[29] Marella M, Devine A, Armecin GF, Zayas J, Marco MJ, Vaughan C (2016). Rapid assessment of disability in the Philippines: understanding prevalence, well-being, and access to the community for people with disabilities to inform the W-DARE project. Popul Health Metr..

[30] Crimmins EM, Kim JK, Solé-Auró A (2011). Gender differences in health: results from SHARE, ELSA and HRS. Eur J Public Health..

[31] Cambois E, Robine JM, Romieu I (2005). The influence of functional limitations and various demographic factors on self-reported activity restriction at older ages. Disabil Rehabil..

[32] Payne CF, Mkandawire J, Kohler H-P (2013). Disability Transitions and Health Expectancies among Adults 45 Years and Older in Malawi: A Cohort-Based Model. PLOS Med..

[33] Cambois E, Duthé G, Soura AB. Living healthy in Ouagadougou: gender differences in the years lived in poorer health and disability in the 20-79 age group. Vienna; 2016.

[34] Bennett R, Chepngeno-Langat G, Evandrou M, Falkingham J (2016). Gender differentials and old age survival in the Nairobi slums. Kenya. Soc Sci Med..

[35] Simo Fotso A, Solaz A, Diene M, Nanfosso RT (2018). Human capital accumulation of children in Cameroon: does disability really matter?. Educ Econ..

[36] Robert Wood Johnson Foundation (2009). Education mater for haelth.

[37] Singleton P, Li L (2016). The dynamic effect of disability on marriage: evidence from the social security disability insurance program.

[38] Manting D, Loeve JA. Economic circumstances and union dissolution of couples in the 1990s in the Netherlands. Netherland: Voorburg/Heerlen: Statistics Netherlands; 2004. https://scholar.google.fr/scholar?hl = fr&as_sdt = 0%2C5&q = Economic+circumstances+and + union+dissolution+of+couples+in+the+1990s + in+the+Netherlands&btnG=. Accessed 17 Jul 2018.

